# Structural Optimization Design of Microfluidic Chips Based on Fast Sequence Pair Algorithm

**DOI:** 10.3390/mi14081577

**Published:** 2023-08-10

**Authors:** Chuang Wu, Jiju Sun, Haithm Yahya Mohammed Almuaalemi, A. S. M. Muhtasim Fuad Sohan, Binfeng Yin

**Affiliations:** 1 School of Mechanical Engineering, Yangzhou University, Yangzhou 225127, China; chuangstu@yzu.edu.cn (J.S.); mh22012@stu.yzu.edu.cn (H.Y.M.A.); 2 Nantong Fuleda Vehicle Accessory Component Co., Ltd., Nantong 226300, China; 3 Jiangsu Tongshun Power Technology Co., Ltd., Nantong 226300, China; 4 Faculty of Engineering, Department of Mechanical Engineering, University of Adelaide, Adelaide, SA 5000, Australia; asmmuhtasimfuad.sohan@student.adelaide.edu.au

**Keywords:** microfluidic chip, simulated annealing algorithm, fast sequence pair algorithm, structural design, optimization algorithm

## Abstract

The market for microfluidic chips is experiencing significant growth; however, their development is hindered by a complex design process and low efficiency. Enhancing microfluidic chips’ design quality and efficiency has emerged as an integral approach to foster their advancement. Currently, the existing structural design schemes lack careful consideration regarding the impact of chip area, microchannel length, and the number of intersections on chip design. This inadequacy leads to redundant chip structures resulting from the separation of layout and wiring design. This study proposes a structural optimization method for microfluidic chips to address these issues utilizing a simulated annealing algorithm. The simulated annealing algorithm generates an initial solution in advance using the fast sequence pair algorithm. Subsequently, an improved simulated annealing algorithm is employed to obtain the optimal solution for the device layout. During the wiring stage, an advanced wiring method is used to designate the high wiring area, thereby increasing the success rate of microfluidic chip wiring. Furthermore, the connection between layout and routing is reinforced through an improved layout adjustment method, which reduces the length of microchannels and the number of intersections. Finally, the effectiveness of the structural optimization approach is validated through six sets of test cases, successfully achieving the objective of enhancing the design quality of microfluidic chips.

## 1. Introduction

Microfluidic technology is a scientific and technological system that integrates fundamental operational units, such as sample preparation, reaction, separation, and detection, into a chip, automating the analysis process [1,2,3]. Microfluidic chips serve as the primary platform for microfluidic technology, utilizing microfluidic channels and reaction chambers to process and manipulate small liquid volumes. Compared with traditional technology platforms, microfluidic chips enable faster completion of tasks that originally took hours, such as separation, isolation, and chemical and biological reactions, resulting in significantly improved detection efficiency [4,5,6].

**Figure 1:**
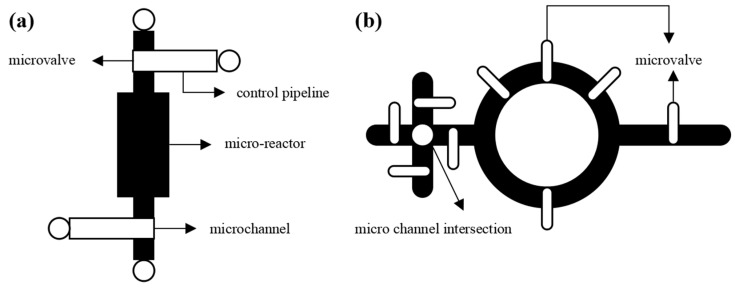
A device with microvalve structure: (**a**) schematic diagram of a chip reaction device; (**b**) schematic diagram of hybrid device.

The structure of a microfluidic chip primarily consists of a fluid layer and a control layer [7,8]. The fluid layer facilitates the reaction process of the experimental reagent. In contrast, the control layer connects to external pressure sources to regulate the flow and stoppage of the reagent within the fluid layer. The connection between the fluid layer and the control layer is established through microvalves, which serve as connectors and can also form devices within the microfluidic chip [9,10]. Figure 1 shows the chip reaction device and a hybrid device containing microvalves. Microvalves will be placed at the crossing point when microchannels cross due to the need for experiments. A typical microfluidics chip can be integrated into hundreds of microvalves. Wiring between microvalves and control ports is required. A large number of microvalves will make wiring difficult. Due to the fact that channel crossing is not allowed in the control layer, the number of microvalves is huge. Once the position of the arrangement is incorrect, it will lead to malfunctions in the control layer. Therefore, it is necessary to reduce the number of microvalves and place them in appropriate positions [11,12,13]. Meanwhile, when the microchannel length is too long, the strong output of an external mechanical pump will affect the execution efficiency and time accuracy of biochemical experiments [14,15,16]. Secondly, when the area of microfluidic chips is too large, it will also cause a waste of raw materials and increase manufacturing costs [17,18,19]. Hence, the number of microchannel intersections, the microchannel length, and the chip area are critical indicators for evaluating the quality of chip structural design.

Reference [20] proposed a component extension method that introduces heuristic diagonals in component extension. References [21,22] proposed an arbitrary angle routing algorithm. In response to storage issues, References [23,24] proposed a distributed channel storage method. References [25,26,27] proposed a weighted sum algorithm for the total length of microchannels. References [28,29] proposed a chip synthesis method for minimizing microvalves. However, in the above research, the layout of components and the routing of microchannels were considered separately, ignoring the interaction between the two, resulting in a decrease in quality and execution efficiency. To consider the interaction between the component layout and runner routing stage, Reference [30] proposed an effective layout and routing algorithm which allows iterative layout adjustment based on feedback information during micro runner routing. It uses a simulated annealing algorithm in the component layout stage, a negotiation-based routing algorithm in the routing stage, and iterative layout adjustment based on micro runner routing information. However, this method’s convergence rate is slow, and the convergence quality is uneven.

Based on a Monte Carlo iterative solution strategy, the simulated annealing algorithm is a stochastic optimization algorithm commonly employed for combinatorial optimization problems [31,32]. Drawing inspiration from the annealing process of solid materials in physics, this algorithm provides an effective approximate solution for issues with non-deterministic polynomial (NP) complexity [33,34,35]. The traditional sequence pair algorithm is a classical layout representation method in the field of electronic design automation. It provides a coding method for the layout scheme of a given device set, which can speed up the enumeration and calculation of layout schemes [36,37].

Focusing on the goal of microfluidics chip structure optimization, this paper uses the improved Fast Sequence Pair (FAST-SP) algorithm to generate the initial solution of the simulated annealing algorithm, improves the cooling speed function of the simulated annealing algorithm, and improves the Rate of convergence of the simulated annealing algorithm. Subsequently, the layout of devices and the routing of microchannels are integrated through layout adjustment using a chip layout quality evaluation function, which reduces the length of microchannels and the number of intersections. Finally, the comparison of data from six experiments demonstrates that the optimization algorithm proposed in this paper effectively enhances the design quality of microfluidic chips.

## 2. Structure Optimization of a Microfluidic Chip

### 2.1. Structural Modeling of Microfluidic Chips

1. Definition of a structural optimization problem

Problem input: experimental process, device summary, device connection relationship, and design constraints.

Problem output: design results, including device layout and microchannel routing results.

Optimization objects: chip area, microchannel length, and number of microchannel intersections.

Design goal: minimize the weighted sum of the chip area, the length of microchannels, and the number of microchannel intersections.

2. Structural design modeling

**Figure 2:**
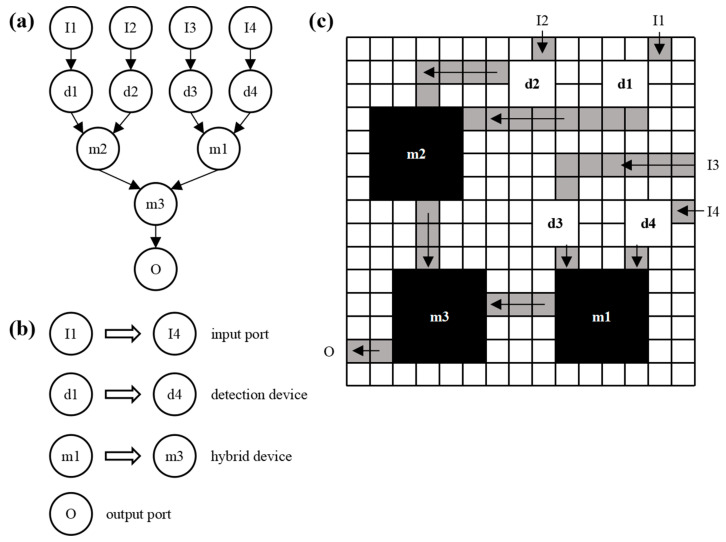
Structural design modeling of microfluidic chips: (**a**) experimental sequence diagram; (**b**) device summary; (**c**) channel wiring diagram of chip device layout.

The structural design of microfluidic chips mainly includes device layout, channel routing, and device layout adjustment [38,39]. Figure 2 is a structural design modeling diagram of the microfluidic chip, and Figure 2a is an experimental sequence diagram showing the observed reaction’s specific flow and the device’s connection relationship. Each node represents a particular operation, such as detection, mixing, etc. Figure 2b summarizes the experimental apparatus. Figure 2c is a schematic diagram of the device layout and channel routing of the microfluidic chip, where the gray channel is the microchannel of the chip.

### 2.2. Algorithm Design

**Figure 3:**
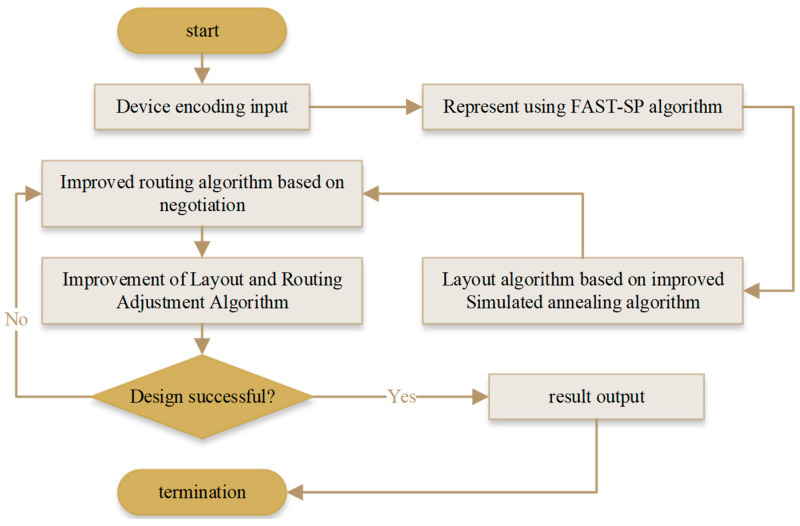
Algorithm design flow chart.

In this paper, the FAST-SP algorithm [40,41,42] represents the initial setting scheme of the device layout. The exact position of each device on the microfluidics chip is calculated through the improved simulated annealing algorithm. Then, the chip layout design scheme is output according to the change process of heating, isothermal, cooling, and search strategies. As shown in Figure 3, the algorithm design process is as follows.

Upon completion of the layout, wiring the microchannels directly would result in experimental redundancy. To address this issue, the devices intended for connection are sequentially linked end to end, utilizing line segments as microchannel lengths in accordance with the experimental sequence diagram. The number of microchannel intersections can be determined by counting the intersection points between these line segments. Once the routing phase is finalized, the optimized layout adjustment algorithm can be employed to rectify the device layout and microchannel routing. The optimized layout adjustment algorithm preserves the initial sequence generated by FAST-SP, but adjusts the component spacing to allow for more routing possibilities. The objective is to eliminate unnecessary microchannel intersections, minimize the number of microvalves, and reduce the overall length of microchannels. Compared with the traditional sequence pair algorithm, the FAST-SP algorithm reduces the complexity of decoding time. After the longest common subsequence (LCS) algorithm is integrated, it speeds up the time to evaluate the layout of sequence pairs, making it more practical. Given a sequence pair, the structure’s starting point, the device’s width and height, and the layout direction can be obtained, and then a configuration can be generated. The starting point refers to the position where the first device is placed. When calculating the relative position between devices, the device’s size is required, and the layout direction is conducive to minimizing the area of the chip. This paper adopts the layout scheme from bottom left to top right.

It is relevant to generate a layout from sequence pairs and find the longest weighted common subsequence in two sequence pairs. Determining the x coordinate of each device is equivalent to calculating LCS (S_X_, S_Y_), choosing the y coordinate of each device is equivalent to calculating LCS (S_X_^R^, S_Y_^R^), and S_X_^R^ is the inverse sequence of S_X_ (Table 1 and Table 2). For the device constraint relationship, each sequence unit in the sequence pair (S_X_, S_Y_) represents each device. Given two devices, a and b, in the sequences S_X_ and S_Y_, if a is before b, then a is on the left of b; in sequence S_X_, if a is before b, and in sequence S_Y_, if a is after b, then a is above b.

**Figure 4:**
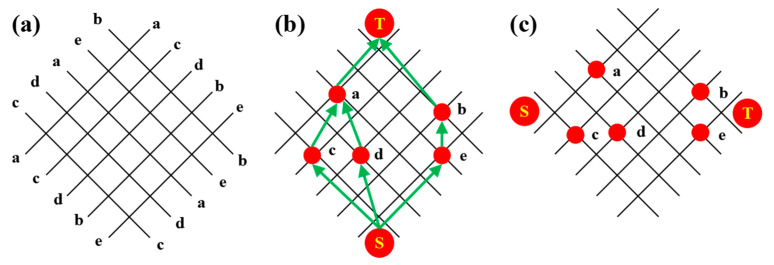
Sequence pair layout: (**a**) skew network of sequence pairs; (**b**) layout vertical constraint diagram; (**c**) layout horizontal constraint diagram.

As shown in Figure 4, given a set of sequence pairs, the grid represents their corresponding constraint relationships. Draw a grid graph of n × n, mark the grid lines from top to bottom with a positive sequence, and mark the grid lines from left to right with an inverse arrangement. Rotate −45° to obtain the oblique grid of sequence pairs <a c d b e> and <c d a c b>. Given this group of sequence pairs, the layout vertical constraint graph (VCG) and layout horizontal constraint graph (HCG) can be obtained according to the start and endpoints.

Lines 1–2 are used to initialize the width of n devices. Line 3 searches for the longest weighted common subsequence of S_X_ and S_Y_ and calculate the x coordinate of each device. Lines 4–5 initialize the height of n devices. Row 6 is used to obtain the reverse-order column S_X_^R^ of S_X_. Line 7 calculates the y-coordinate of each device according to the longest weighted common subsequence of S_X_^R^ and S_Y_.

Lines 1–2 represent vector blocks_order, which records the index of each device in S_2_. Line 3 initializes the vector length to 0, saving the maximum length of each device. Lines 4–5 indicate that the variable block is equivalent to the current device in S_1_. Line 6 indicates that the index is the index of the current device in S_2_. The block’s position in Line 7 is defined as the position not occupied by other devices at first. Lines 9–12 update the lengths. The last quantity of Line 13 stores the total determined length.

(1) Calculate the weighted LCS width of two sequences, S_1_ and S_2_, and use the vector block order to record the index of each device in S_2_.
(2) Initialize vector lengths to 0 to store each device’s maximum quantity (length or width).
(3) The variable block is defined as the current device in S_1_, and the index is the index of the current device in S_2_. All the items on the left of the block are arranged into intervals with the length of the block, and the block is then placed.
(4) The total length of the updated layout is length, and length [n] indicates the full length after n devices are determined.
(5) It is updated if the length [j] exceeds the current length.

Extract the LCS algorithm when S_1_ = S_X_, S_2_ = S_Y_, and weights = width, which can define the x coordinate of the layout. Extract the LCS algorithm when S_1_ = S_X_^R^, S_2_ = S_Y_, and weights = heights, which can define the y coordinate of the layout.

### 2.3. Layout Calculation Optimization

The traditional simulated annealing algorithm has the problem that the parameters greatly impact the experimental results, and the convergence quality is not high when dealing with layout problems [43,44]. For this reason, this paper improves the simulated annealing algorithm, and the main improvement strategies are divided into two types: ① changing the factors of the algorithm itself; ② changing the search strategy, speeding up the search, and improving the search quality.

(1) Change of algorithm cooling function

The control methods of simulated annealing in temperature cooling are divided into rapid cooling mode (RSA) and general cooling mode (CSA).

RSA: T = T_0_/log(1 + N); CSA: T = qT_0_ + k, where q is the cooling rate and k is a constant.

Since the initial cooling rate of RSA and CSA is too fast to obtain the global optimal solution, this paper improves the algorithm from the cooling rate and proposes an improved simulated annealing algorithm (ISA), as shown in Formula 1:

(1) $$T=T_{0}\times e^{\left(-N^{3}/T_{0}^{2}\right)}$$

T_0_ is the temperature at the initial time, N is the number of iterations required by the algorithm in the external cycle, and T is the current temperature.

**Figure 5:**
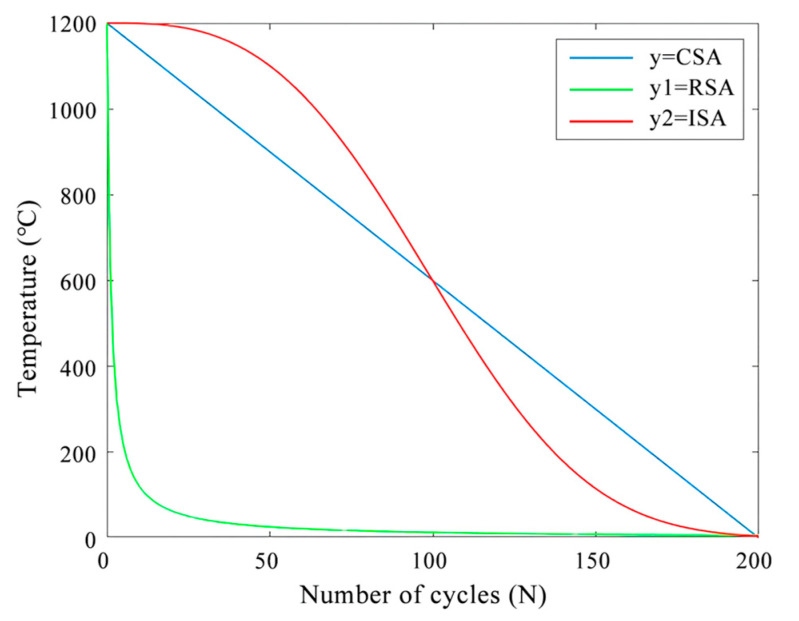
Cooling function curve, where CSA is the function curve of the available cooling mode, RSA is the function curve of the rapid cooling mode, and ISA is the function curve of the improved simulated annealing algorithm.

As shown in Figure 5, compared with RSA and CSA, the ISA algorithm proposed in this paper decreases slowly in the high-temperature stage, achieving a global search of the algorithm, which is more conducive to generating the optimal solution.

The slow cooling rate can easily cause the slow global Rate of convergence. The convergence rate can be accelerated by combining the priority scheme of the FAST-SP algorithm with the ISA algorithm. The improvement methods are as follows:

(1) Use reverse transformation to select two device units and reverse all units between the two units.
(2) Select three device units and switch the unit between the two device units to the back of the third unit.

(2) Change of algorithmic search strategy

(1) Expand the recording function. During the implementation of the algorithm, the particularity of its selection probability may cause the problem that even if the optimal solution is generated, it may be abandoned. The extended recording function can save the optimal solution in this cycle for comparison with the results generated later.

Add a memory matrix (I) and a function (F) in the simulated annealing algorithm. Initially, there is an element i_0_ in I, and F = f (i_0_). When generating a new solution, each time a new solution (j) is obtained, compare F with f (j). If f (j) < F, let F = f (j), and store j in I. After the algorithm is completed, the optimal solution is compared with the solution recorded in I to select the optimal solution as the final solution of the algorithm.

(2) For the solution of the initial solution, the simulated annealing algorithm with the recording function is used first. After the trial run of the entire algorithm is completed, the final result obtained is searched locally until the local algorithm search is conducted. Then, the final solution, namely the optimal solution, is output.

### 2.4. Routing Algorithm Optimization

The wiring of microfluidic chips can be divided into two stages. In the first stage, the A* algorithm is mainly used for routing because there is no microchannel intersection [45,46,47]. If the wiring is successful, the result will be output. If the wiring fails, the second stage will be carried out. In the second stage, the microchannels are allowed to generate intersections during routing. Then, the routing is carried out through the improved A* algorithm, adding additional generation value to the grid. Whether it is the first or second stage, the existing algorithms use routing based on the experimental response order [48,49]. Although the extra generation value of the routing grid can be iterated to obtain the routing scheme, the routing quality is not high.

**Figure 6:**
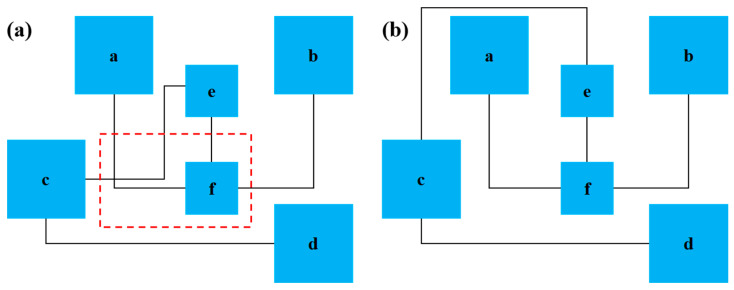
A schematic diagram of two types of wiring results: (**a**) there is an intersection; (**b**) no intersection exists.

As shown in Figure 6a, the blue box is a device, and the black line is a microchannel. When six devices are wired, a microchannel intersection will be generated, and the experimental reaction sequence is af, bf, and dcef. As shown in Figure 6b, the intersection disappears when the microchannel of connector f is wired first. It can be seen that key devices are essential to the results of microchannel wiring, and the probability that key devices can be wired in this area is higher than in other areas.

**Figure 7:**
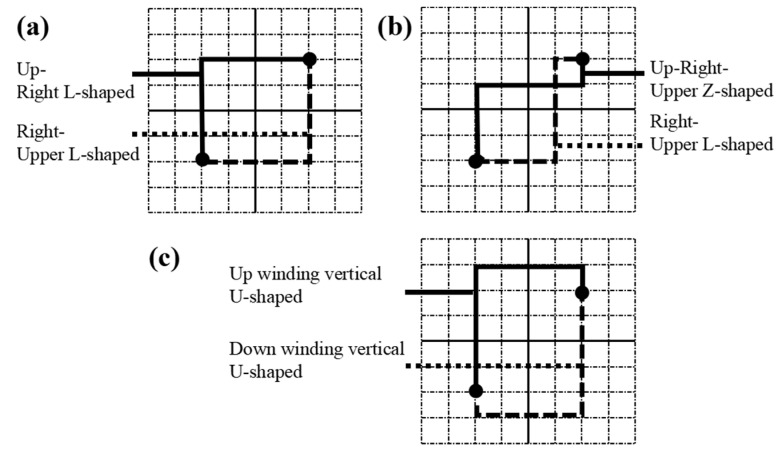
Wiring mode: (**a**) l-shaped wiring; (**b**) z-wiring; (**c**) u-shaped wiring.

For this reason, in the wiring stage of a microfluidic chip, this paper gives priority to the wiring of devices with multiple microchannels, defines the high wiring area (in the red dotted box) to distinguish it from other regions, and then wires the chip microchannels based on the improved wiring algorithm. In addition, the main purpose of wiring is to shorten the length of the microchannel and speed up the biochemical reaction time in the microchannel. To this end, three types of wiring modes, as shown in Figure 7, can also be selected to reduce the number of cross-points in microchannels.

### 2.5. Adjustment Algorithm Optimization

The previous device layout and microchannel routing results must be adjusted in the layout adjustment phase. Fine-tune the devices while keeping the relative position of the devices unchanged in the congested area; that is, keep the sequence pair order intact. The congestion area contains most devices, microchannels, and microchannel intersections. After adjustment, the next iteration will be carried out if the layout and wiring results are better than the previous work. They will be discarded if they are not as good as the earlier work [50,51,52]. However, this method only adjusts the congestion area, and the adjustment spacing is too small, which affects the overall layout quality to a certain extent and may result in the failure of layout adjustment.

For this reason, when adjusting the device layout and microchannel routing, this paper adjusts the devices in all areas where there are microchannel intersections and multiple channels, moving two units of length to the left, down, or right while maintaining the same relative position and moving one unit of measurement for devices in areas where there is no microchannel intersection. The purpose is to provide space for machine and microchannel adjustment in other congested areas and ensure the algorithm can optimize the layout results without defects. If the result after adjustment is better than the previous scheme, the optimal solution will be output.

### 2.6. Improved Algorithm Flow

On the premise of device set and device connection relationships and with the device layout scheme obtained by the FAST-SP algorithm as the input, the following conditions are defined:

1. The distance between device mi and device mj on its right or above is defined as rx and axe.
2. The width and height of all device set spacing are defined as WX and WY. The constraint condition of elements between WX and WY is [e_min_, e_max_]. WX and WY form the initial solution S.
3. In the simulated annealing algorithm, the initial temperature is defined as T, the number of external cycles is N, the end temperature is T_end_, the current temperature is T_0_, the chain length is L, and the quality function for evaluating the chip layout is E (S).

The algorithm flow is:

(1) Initialize WX and HY: e_min_ < rx, rx < e_max_.
(2) Set the state variables S = (S_X_, S_Y_, WX, HY) and the initial temperature T. When the initial test temperature exceeds the minimum temperature, the iteration starts.
(3) Adjust the state variable S -> S_1_, randomly generate the variables rx^1^ and ax^1^, and compare them with e_min_ and e_max_. When rx^1^ < e_min_, let e_min_ = rx^1^; when rx^1^ > e_max_, let e_min_ = rx^1^. The ax^1^ is obtained in the same way.
(4) Utilize Metropolis guidelines [53,54,55]:

(2) $$df=E\left(S^{\prime }\right)-E\left(S\right)$$

(3) $$P=\left\{\begin{array}{c} \begin{array}{cc} 1, & df<0 \end{array}\\ \begin{array}{cc} \exp\left(-df/T\right), & df\geq 0 \end{array} \end{array}\right.$$

(4) $$P^{\prime }=\left\{\begin{array}{c} \begin{array}{cc} 1, & df<0 \end{array}\\ \begin{array}{cc} \exp\left(-df/\left(T_{0}\times e^{\left(-N^{3}/T_{0}^{2}\right)}\right)\right), & df\geq 0 \end{array} \end{array}\right.$$

If df < 0, the newly generated layout result will be accepted; otherwise, the new layout result will be obtained with probability exp (−df/T).

(5) Cool down. Use the new cooling rate function to cool down. Stop iterating and output the current result if T_0_ exceeds the end temperature.

Formula (4) is obtained by combining Formulas (2) and (3). The improved simulated annealing algorithm proposed in this paper converges quickly and can obtain a better solution when facing more devices.

For example, the parameters are e_min_ = 3, e_max_ = 5, T = 10,000, T_end_ = 10^−4^, and L = 200, and the cooling rate is 0.95.

Evaluate the chip layout quality functions:

(5) $$E\left(S\right)=\alpha A+\beta B+\gamma C+\theta C^{2}$$

where A is the chip layout area, B is the number of microchannel intersections, C is the length of the microchannel segment, and C^2^ is the square of the total segment. The main purpose is to minimize and enhance the length of the microchannel. Set the weight values of α to 1, β to 300, γ to 20, and θ to 0.001.

After the layout is completed, the A* algorithm can be used to find the shortest path after the layout. The input of the algorithm includes the following:

① Non-negative edge weight graph G (WX, HY).
② A starting source node s.
③ One target end node t.

Then, two sets, M and N, are introduced, where M contains the point of the shortest path that has been found and the length of the shortest route, and N is the point of the shortest path that has not been found and the distance from the point to the source node.

Initialize the two sets M and N, find the shortest path point from the N set, add it to the M set, then update the devices in the N set, iterate circularly until the end of the traversal, and find the best routing result of the microfluidic chip.

## 3. Experimental Results and Analysis

We compared the proposed comprehensive optimization algorithm with the manual layout and existing algorithms [56] to verify the effectiveness of the proposed algorithm. Among them, the existing algorithm uses the basic simulated annealing algorithm, and this paper optimizes on this basis. Manual layout refers to a layout that has not undergone algorithm-optimization adjustments. The algorithm in this article was implemented using C++ programming language. The experimental platform was a 64-bit Windows server, configured with a 2.40 GHz Intel processor and 32 GB of memory to implement.

As shown in Table 3, in order to better compare the performance of algorithms, this paper selects six groups of test examples that can be completed without control layer iteration. Among them, the number of devices in the polymerase Chain reaction (PCR) group is 16, the number of devices in the InVitro-1~InVitro-3 groups is 30, 45, and 60, and the number of devices in the ProteinSplit-1 and ProteinSplit-2 groups is 30 and 66, respectively. The experimental results show that the integrated optimization algorithm in this paper reduced the chip area by 17.6%, the microchannel length by 20.9%, the number of microchannel intersections by 24.0%, and the convergence time was reduced by 16.9% on average, with the optimization ratio of PCR reaching 47.3%, compared with existing algorithms. From the improvement percentage, it can be concluded that when the number of devices was 45, the comprehensive optimization algorithm in this paper was optimal.

**Figure 8:**
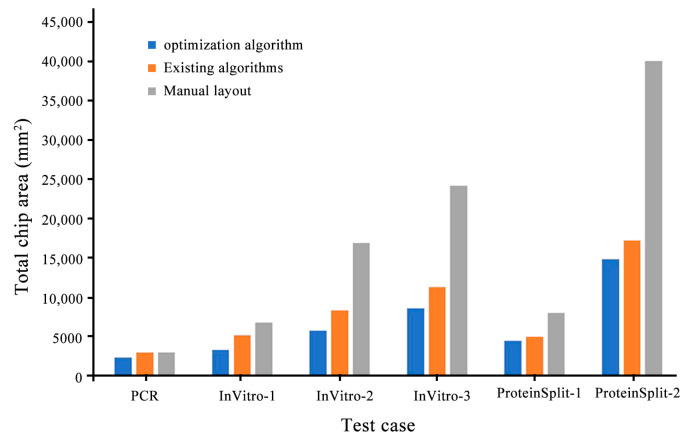
Comparison of three types of algorithms in total chip area.

**Figure 9:**
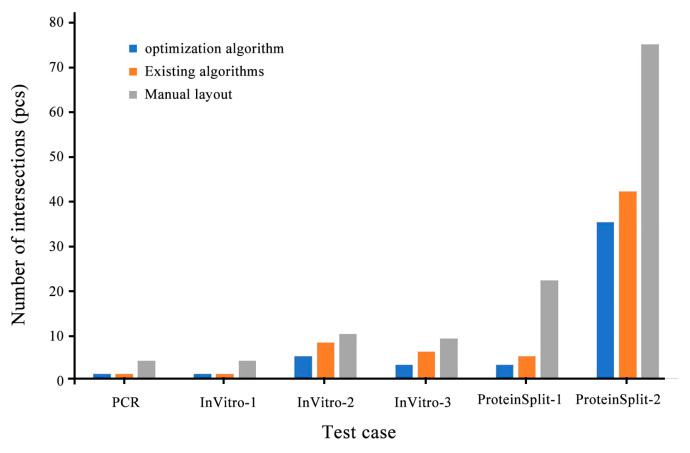
Comparison of three types of algorithms on the number of intersections.

**Figure 10:**
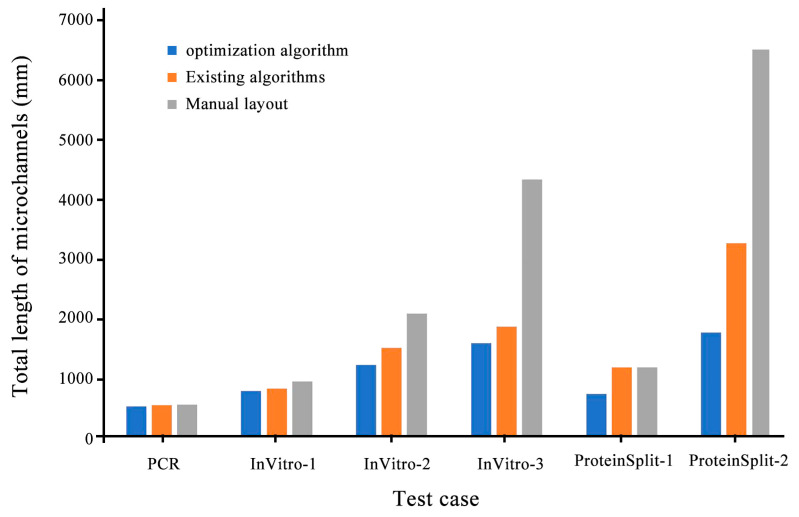
Comparison of 3D algorithms on the total length of microchannels.

Figure 8, Figure 9 and Figure 10 compare the integrated optimization algorithm, the existing algorithm, and manual layout in the total chip area, the number of microchannel intersections, and the total length of microchannels. The figure shows the effectiveness of the integrated optimization algorithm in the structural design of microfluidic chips and the necessity of the mechanical structural optimization design of microfluidic chips.

The experimental results show that the optimization algorithm proposed in this paper can meet the automatic design requirements of microfluidic chips. The advantages of the optimization algorithm in the structural design of microfluidic chips are verified through the reduction of the chip area, the shortening of the microfluidic channels, and the reduction of the number of intersections.

## 4. Conclusions

This FAST-SP algorithm addresses the challenges in chip layout design. By leveraging the FAST-SP algorithm, the proposed method accelerates the convergence rate of the simulated annealing algorithm, enhances its cooling rate and search strategy, mitigates the impact of parameters on the outcomes, and improves convergence quality. This approach targets the limitations of existing layout design methods for microfluidic chips, which often lack global optimization capabilities. Additionally, it enhances the routing method to minimize the number of intersections between microchannels. The key contribution is strengthening the interaction between microchannel routing and device layout. Six test cases were reconducted to evaluate the proposed algorithm’s effectiveness, with chip area, microchannel length, and the number of intersections serving as optimization objectives. The results clearly state the superiority of the optimization algorithm presented in this paper compared with existing design methods in these three areas. Notably, the experimental data reveal that when the number of devices reaches 45, the algorithm achieves the optimal improvement percentage, highlighting its capability to obtain optimal solutions and exhibit robustness.

Through enhancements, the optimization algorithm achieved an average reduction of 17.6% in the total chip area, 20.9% in the overall microchannel length, 24.0% in the number of microchannel intersections, and 16.9% in the convergence time. These quantitative results unequivocally validate the effectiveness of the proposed optimization algorithm in optimizing the structure of microfluidic chips and enhancing their design quality paper, introducing an improved simulated annealing algorithm that builds upon the FAST-SP.

## Figures and Tables

**Table 1:** Sequence pair algorithm.

Input: sequence pair (S_X_, S_Y_), width (length) of n devices, width [n] (heights [n]).	
Output: x (y) coordinates x_coords (y_coords), the W (H) dimension of the layout structure.	
1. for(i = 1 to n)	
2. weights [i] = widths [i]	//Weight of device-width
3. (x_coords, W) = LCS (S_X_, S_Y_, weights)	//X coordinate, total width W
4. for(i = 1 to n)	
5. weights [i] = heights [i]	//Weight of device height
6. S_X_^R^[i] = S_X_[n + 1 − i]	//Reverse S_X_
7. (y_coords, H) = LCS(S_X_^R^, S_Y_, weights)	//Y coordinate, total height H

**Table 2:** LCS algorithm.

Input: sequences S_1_ and S_2_, weights of n devices [n]	
Output: position of each module, total length L	
1. for(i = 1 to n)	
2. block_order[S_2_[i]] = i	//Index of each device in S_2_
3. lengths[i] = 0	//Total length initialization of all devices
4. for(i = 1 to n)	
5. block = S_1_[i]	//Current device
6. index = block_order[block]	//Index of current device in S_2_
7. positions[block] = lengths[index]	//Calculate the position of the device
8. t_span = positions[block] + weights[block]	//Determine the current fast length
9. for(j = index to n)	
10. if(t_span > lengths[j])	
11. lengths[j] = t_span	//The length of the current device replaces the former
12. else break	
13. L = lengths[n]	//Total length

**Table 3:** Comparison of experimental results.

Test Case	Chip Area (mm^2^) Existing Algorithm/Optimization Algorithm	Microchannel Length (mm) Existing Algorithm/Optimization Algorithm	Microchannel Intersection (pcs) Existing Algorithm/Optimization Algorithm	CPU Time (s) Existing Algorithm/Optimization Algorithm	Percent Improvement (%)
PCR	2958/2850	522/509	1/1	42.7/22.5	3.6
InVitro-1	5110/3906	802/765	1/1	84.1/63.7	23.5
InVitro-2	8232/5688	1485/1203	8/5	179.7/170.9	30.9
InVitro-3	11,187/8460	1864/1568	6/3	301.3/245.6	24.3
ProteinSplit-1	4914/4422	1162/713	5/3	114.0/106.9	10.0
ProteinSplit-2	17,030/14,690	3247/1749	42/35	528.0/527.7	13.7

## Data Availability

Data sharing is not applicable to this article.
